# Psychological research in eating disorders/disordered eating in type 1 diabetes: A systematic review using the NIH stage model for behavioural intervention development

**DOI:** 10.1111/dme.70369

**Published:** 2026-05-21

**Authors:** Heather L. Stuckey, Debbie Cooke, Amy C. Knehans, Timothy C. Skinner

**Affiliations:** ^1^ Department of Medicine Pennsylvania State University College of Medicine Hershey Pennsylvania USA; ^2^ Department of Health & Medical Sciences University of Surrey Guildford UK; ^3^ Department of Library Sciences Pennsylvania State University College of Medicine Hershey Pennsylvania USA; ^4^ Department of Health Psychology Australian Centre for Behavioural Research in Diabetes Melbourne Victoria Australia; ^5^ Institute for Health Transformation, Deakin University Geelong Victoria Australia; ^6^ Department of Health and Caring Sciences Western Norway University of Applied Science Bergen Norway

**Keywords:** psychosocial, systematic reviews, type 1 diabetes

## Abstract

**Aims:**

Eating disorders in type 1 diabetes are 2.4 times more likely to develop than those who do not have diabetes. The aim was to conduct a systematic review on psychological interventions in IRDEB (insulin‐related disordered eating behaviour)/eating disorders with adults who have type 1 diabetes. The review describes the type and quality of studies with control/comparison groups, as well as qualitative research, and synthesises the research according to the NIH Stage Model of Behavioural Intervention Development. Classification according to the Model allows for discussion of gaps and recommendations for further research.

**Methods:**

The methodology for this research followed PRISMA guidelines, with inclusion criteria of: (1) type 1 diabetes; (2) adults (age ≥18); (3) behavioural and psychological interventions in eating disorders or disordered eating; (4) primary outcome being psychological, (5) conducted from 2014 to February 2024; (6) in English; (7) full‐text, peer‐reviewed articles; AND (8) quantitative trials with control or comparison groups OR (9) qualitative research.

**Results:**

From a total of 28 studies related to type 1 diabetes and psychological outcomes, nine studies were found to be relevant to IRDEB and eating disorders. Although studies of disorders have risen in publication, there was only one completed full RCT since 2014, which met the inclusion criteria, the *Diabetes Body Project.* Methodological designs for the nine studies included six qualitative studies and three experimental studies. Six studies were categorised as Stage 0, one as Stage 1, one as Stage II and one as Stage III. Two studies included participants with ‘diabulimia’ alone, and the seven remaining studies combined eating disorders and eating disorder symptoms (e.g., ‘diabulimia’, anorexia, binge‐eating, bulimia).

**Conclusions:**

With the limited research that is available, and only one recently completed RCT, the opportunities for research in IRDEB/eating disorders are capacious. There is a need to tailor existing eating disorder interventions to T1D and to continue to investigate the efficacy (Stages II and III), and effectiveness (Stages IV and V), keeping in mind the unique needs and conditions under which IRDEB and eating disorder symptoms emerge.

**What‘s new?**

**What is already known?**

- Type 1 diabetes is associated with a 2.5 times increased risk for IRDEB (insulin‐related disordered eating behaviour) and eating disorders. Treating adults with both IRDEB/eating disorders and diabetes presents a dual challenge that necessitates a comprehensive approach, but little research exists.

**What this study has found?**

- Only one project, The Diabetes Body Project, completed an RCT in Stage III that demonstrated efficacy in the ‘real world’ of type 1 diabetes treatment in adults.

**What are the implications of the study?**

- More RCTs with qualitative data and longitudinal trials of IRDEB/eating disorders need to be undertaken, supported by funding mechanisms for researchers to continue to advance their research forward in the NIH Stage Model. The need for evidence‐based, implementable interventions is growing to help adults with type 1 diabetes IRDEB/eating disorders.

## 1 INTRODUCTION/BACKGROUND

The daily demands of managing type 1 diabetes, with its consequential focus on carbohydrate counting, potential impact on body shape and the ready availability of weight management through insulin, are among the factors that place young adults and adults at increased risk of developing a range of dysregulated eating patterns.^1^, ^2^, ^3^ Diagnosed eating disorders in type 1 diabetes include anorexia nervosa, bulimia nervosa, binge‐eating disorder. ‘eating disorder not otherwise specified’ (EDNOS),^4^ or ‘other specified feeding or eating disorder’ (OSFED).

Eating disorders are thought to be close to two and a half times more common in people with type 1 diabetes than in people without, with a prevalence ranging from 8% to 36%.^5^, ^6^, ^7^ Binge‐purge spectrum symptoms, which generally fall under the purview of subclinical eating disorders, are the most reported eating disorder symptoms in people with type 1 diabetes^8^; with no differences in incidence of anorexia nervosa in those with type 1 diabetes and those without diabetes.^7^ These subclinical eating disorder symptoms (disordered eating behaviours) do not meet criteria for a clinical eating disorder but cause increased stress, loss of control and difficult feelings like guilt.^9^ Individuals with type 1 diabetes have the unique weight regulating and calorie purging tool, intentional insulin restriction which induces glycosuria, colloquially referred to as ‘diabulimia’.^10^ There are no meta‐analytic data that would provide an estimate of the prevalence of intentional insulin restriction. In the latest recommendations for consistent terminology, we used IRDEB (insulin‐related disordered eating behaviour)/eating disorders in this systematic review to refer to adults with type 1 diabetes who have dysregulated eating patterns.^11^ If the author of the manuscript used a different terminology, such as ‘diabulimia’, we did not change their wording of the term.

While every type of eating disorder can be life‐threatening, IRDEB/eating disorders in type 1 diabetes are especially dangerous. Adults with type 1 diabetes and anorexia nervosa are at risk for renal failure and can have more frequent and recurring hospitalisations from severe low blood sugars.^12^ Bulimia and/or binge‐eating can cause a nearly constant state of dangerous ketosis or diabetic ketoacidosis with blood sugars frequently at dangerously high levels. ‘Diabulimia’ increases a person's risk of developing long‐term complications, including blindness, neuropathy, gastroparesis, kidney failure and a greater mortality risk.^8^, ^13^, ^14^

Not only are there substantial biomedical risks that arise from living with IRDEB/eating disorders, but emotional well‐being challenges that form the behaviours, affect and cognition of the person living with it. The primary goal of an IRDEB/eating disorder intervention is to facilitate recovery from the disturbed eating patterns that affect the biomedical outcome (i.e., weight, HbA1c, diabetic ketoacidosis),^1^ with psychological treatment as a primary line of defence. The goal is to relieve the main and associated symptoms, improve and maintain a normal weight, and enhance quality of life.^15^ Psychological interventions (e.g., acceptance and commitment therapy)^16^, ^17^ are used in IRDEB/eating disorder treatments since they address psychological factors involved in onset and maintenance. The purpose of this systematic review is to focus on the primary outcome of psychological interventions, rather than biomedical outcomes.

Altabas et al.^18^ and D'Silva et al.^19^ demonstrate that the best‐evidenced interventions to date (notably cognitive dissonance–based programmes which aim to reduce thin‐ideal internalisation and body dissatisfaction) ‐consistently reduce eating disorder risk factors and ‐diabetes‐specific eating pathology. This necessitates early detection, multidisciplinary care and improved diagnostic frameworks. Addressing disordered eating requires integrated, developmentally tailored and longer‐term interventions that explicitly target insulin manipulation alongside psychological risk factors, supported by better screening tools, family/peer involvement and robust trials. In a much earlier systematic review, inpatient therapy appeared to be the most effective treatment at reducing eating disordered symptoms and glycaemic control; and this had multiple components including cognitive behavioural therapy, psychoeducation and family therapy.^20^

The aim of the systematic review is to describe the type and quality of psychological studies in IRDEB/eating disorders in adults (age ≥18) with type 1 diabetes. The review describes the type and quality of students with control/comparison groups and synthesises the research according to the NIH Stage Model of Behavioral Intervention Development. In addition, we included qualitative studies that informed the development of further research. Classification according to the Model allows for discussion of the state of the current research, along with gaps and recommendations for further stages of research development.

According to their website, NIH Stage Model for Behavioral Intervention Development | National Institute on Aging, the model of behavioural intervention development is composed of six stages: basic science (Stage 0), intervention generation, refinement, modification and adaptation and pilot testing (Stage I); traditional efficacy‐testing (Stage II); efficacy‐testing with real‐world providers (Stage III); effectiveness research (Stage IV) and; dissemination and implementation research (Stage V). Examination of mechanisms of behaviour change is encouraged in every stage of intervention development. Consideration of the intervention's ease of implementation also is encouraged as early as possible in the intervention development process, as well as qualitative research to inform the process. The ultimate goal is to produce highly effective and maximally implementable behavioural interventions that improve health and well‐being. The NIH Stage Model is suited to evaluate the literature in an area of low possible numbers, where meta‐analytic approaches serve little value due to the small number of studies, small number of participants, heterogeneity of participants and the diversity of outcomes likely to be included.

During the last 10 years, there was only one meta‐synthesis of qualitative research and IRDEB experiences,^9^ and a search of primary articles that contained interviews of people with IRDEB,^21^ but no reviews about the psychological outcomes of interventions or qualitative research in IRDEB/eating disorders. We found one review (in multiple sclerosis, not diabetes) that adopted the model to frame the literature for describing future interventions.^22^

Thus, we conducted a systematic review using the NIH Stage Model for Behavioural Intervention Development as our analytical and synthesising framework for psychological research in IRDEB/eating disorders in type 1 diabetes. We also included a unique category of future research and implications, which is not typically included in a systematic review. This allowed us to determine whether researchers recognised the Model by suggesting future work that would move their research to the next level of investigation.

This review of literature may assist healthcare providers who are looking for the most up‐to‐date studies on psychological interventions and how to consider implementing research into real‐world practice. Because areas for future research and directions are included as part of this review, healthcare funding agencies may be able to determine high priority areas for funding in diabetes and IRDEB/eating disorders. Ultimately, people with type 1 diabetes will benefit from learning about research available to those with IRDEB/eating disorders.

## 2 METHODS

### 2.1 Study selection

This systematic review study protocol has been registered on the International Prospective Register of Systematic Reviews (PROSPERO) (CRD42025636451) and can be accessed at https://www.crd.york.ac.uk/PROSPERO/. We employed Preferred Reporting Items for Systematic reviews and Meta‐Analyses (PRISMA) guidelines for the systematic review.^23^ The focus on IRDEB/eating disorders is a subset of a larger psychological outcomes systematic review to be published under separate review. This methods section summarises search strategies for the larger study.

PubMed was the primary search database, using the following MeSH terms: Diabetes Mellitus, Type 1; Psychological Intervention; Depression; Depressive Disorder; and Feeding and Eating Disorders along with relevant keywords. The complete search strategy, including MeSH terms, keywords, Boolean logic, screening criteria and search timeframes, is provided in accordance with PRISMA guidelines (Table S1). We searched additional databases: Cochrane Library, Scopus, PubMed, Web of Science, CINAHL, Embase, PsychINFO and ClinicalTrials.gov.

Inclusion criteria for quantitative, experimental studies were peer‐reviewed publications from research with a control or comparison group on or after 2014 through February 2024, in English, full‐text original research articles, type 1 diabetes and primary outcome being psychological. One of the reasons for including only studies with a control or comparison group is that participation in a study can produce positive outcomes alone; therefore, it is necessary to have a comparator to determine changes in the intervention group from a control.^24^ Indeed, simple recruitment in a clinical trial, independent of any therapeutic intervention, produces improvements in outcomes.^25^ Qualitative publications were included using all the same criteria, except a control or comparison group was not relevant for qualitative studies.

Exclusion criteria were as follows: review articles, non‐English, published before 2014, non‐control or comparison group studies (quantitative), conference abstracts and wrong age group (<18). Systematic reviews related to eating disorders/disordered eating and T1 were saved and manually reviewed to identify additional articles that potentially met inclusion criteria. Eleven studies from these reviews were pulled for screening (three were duplicates); and eight were screened for full‐text review.

In addition to PRISMA, we used the Mixed Methods Appraisal Tool (MMAT) for quality assessment to enhance reporting transparency and methodological consistency.^26^ Due to the diversity of study methods used in this review (both qualitative and quantitative), the MMAT provided specific criteria for evaluating each method, overcoming the challenge of using separate tools. The tool lists two screening questions for all study evaluation regarding the clarity of the research question and the appropriateness of the collected data in answering the research question, to which studies received ‘yes’, followed by five closed‐ended (yes/no/can't tell) methodological quality criteria for five study types (qualitative, quantitative RCTs, quantitative non‐randomised, quantitative descriptive and mixed methods). Each study was independently reviewed by two researchers (DC and TS), with discrepancies resolved by a third reviewer (HS).

**FIGURE 1:**
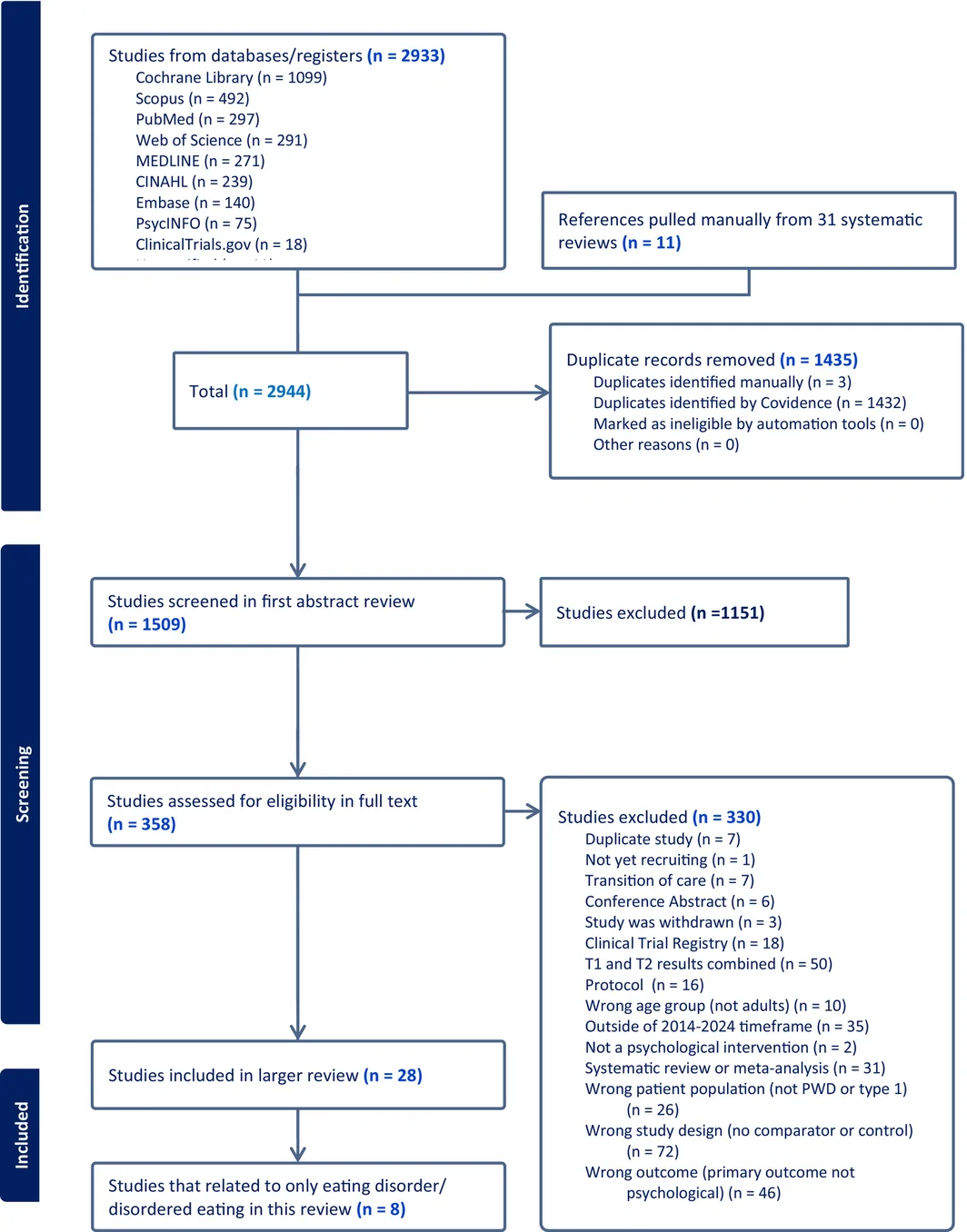
PRISMA flow diagram.

The PRISMA diagram (Figure 1) shows the initial number of records identified by the Cochrane Library (*k* = 1099), Scopus (*k* = 492), PubMed (*k* = 305), Web of Science (*k* = 291), MEDLINE (*k* = 271), CINAHL (*k* = 239), Embase (*k* = 140) and PsycINFO (*k* = 75). ClinicalTrials.gov (*k* = 18) and unspecified sources (*k* = 11). An initial 2941 records were identified. Additionally, we pulled 31 systematic reviews to extract additional references (*n* = 11) that were not identified in the above strategies, for a total (*k* = 2952).

Duplicate records from Covidence were removed (*n* = 1432), as well as manual records (*n* = 3), for a total of 1435 records removed due to duplication. This resulted in 1517 records screened during the first abstract review led by two reviewers (DC and HS) with a tie breaker (TS) using the inclusion and exclusion criteria. 1151 studies were excluded during the first round title abstract review and is marked as ‘studies excluded’, leaving 359 records which were further assessed for eligibility. Figure 1 lists the exclusion reasons and numbers, with more common reasons being: (1) wrong outcome (primary outcome not psychological) (*k* = 59); (2) T1 and T2 results combined (*n* = 52), meaning that we could not determine outcomes for T1 only; (3) wrong study design (no comparator or control for experimental studies) (*n* = 39); (4) outside of 2014–2024 timeframe (*n* = 34); (5) systematic review or meta‐analysis (*n* = 31) or (6) wrong patient population (not people with diabetes or type 1 diabetes) (*n* = 26). Thus, 22 studies from the larger review, related to psychological outcomes in T1D, were included for analysis. We then searched those articles to evaluate those related to only eating disorders/disordered eating, with nine meeting that criterion. Six studies were qualitative, and three were quantitative, experimental designs.

When determining the stage of research, the three authors (HS, DC, TS) reviewed each methods section of the article independently for both the stage of the NIH Model and reasoning as to why the stage was assigned. When consensus was met with all three authors, we concluded the outcome. If consensus was not reached, we made a public inquiry to the Acting Branch Chief, National Institute on Aging at NIH. The NIA was the first to provide a systematic framework for advancing behavioural intervention science, bridging basic research with the development and dissemination of interventions that are scientifically rigorous and scalable.^27^, ^28^

### 2.2 Data extraction

Using Covidence as the software management tool, we imported all references that met the criteria for inclusion. For each included study, two independent co‐authors (HS and DC) reviewed the abstract to determine its advancement to the next stage of full review. At that point, the articles were either advanced with a no or yes. If consensus was not achieved, a third independent reviewer (TS) was assigned to resolve the conflict. The process was repeated for both the abstract review and the full review. After the full review, the following data were extracted from the eligible studies: first author's name, year of publication, country, study design, recruitment method, inclusion and exclusion criteria, sample size; and where relevant for each of the studies extracted: method of randomisation, age, gender, BMI, duration of diabetes, HbA1c, description of the intervention and what the control group received, virtual or online intervention delivery, who delivered the intervention, dropouts in each arm, outcomes collected and method of assessment (e.g. questionnaire tool), means, standard deviations and effect sizes for all relevant outcomes collected, future research advised, recommendations for clinical practice, and a brief conclusion (Table S2) and, for qualitative studies, the themes and sub‐themes identified (Table S3). The mixed methods study combined both qualitative and quantitative approaches (Table S4).

## 3 RESULTS

From the nine studies related to IRDEB/eating disorder, methodological designs included: a pilot RCT^29^ followed by an RCT,^30^ a qualitative participatory action design process,^31^ a concurrent mixed methods study,^32^ and five descriptive qualitative studies^14^, ^33^, ^34^, ^35^, ^36^ (Table 1). Three studies included participants with ‘diabulimia’ alone^14^, ^33^, ^36^ and the six remaining studies combined eating disorders and eating disorder symptoms (e.g., ‘diabulimia’, anorexia, binge‐eating, bulimia). In the quality assessment of the studies (Table 2), all studies scored positively on quality dimensions, two studies had questionable selection of data collection methods as described in the notes section of the table. This indicates that the studies were of generally sound quality.

**TABLE 1:** T1DE studies included in the systematic review, by Stage Model designation.

References	Stage	Design & focus	Study aim	Findings	Future research and implications
Verbist and Condon^14^	0	Concurrent Mixed Methods Exploratory: Prevention	To identify predictors of disordered eating and qualitatively explore links between T1D management and body Image concerns	Young age and negative body image explained 58.8% of disordered eating variance, while body weight and social networking negatively contributed to poor body image (29.9%). Themes: Feeling a lack of control over body weight, limitation in clothing options and discomfort towards medical equipment	These findings could be used as a sound foundation for prevention programs targeting an increase in body image satisfaction among the T1D population
Coleman and Caswell^15^	0	Qualitative Exploratory	To conduct an exploratory analysis into the views and experiences of people with lived experience of *diabulimia*	Themes: Concerns about weight, difficulty coping with diabetes, past trauma, and the importance of relationships. Experiences with health professionals were overwhelmingly negative	This would provide HCPs with an increased understanding of *diabulimia* and pave the way for future psychological interventions, specifically targeting trauma and beliefs about weight and appearance. Interventions should focus on helping individuals to cope with a long‐term condition
Hastings and McNamara^16^	0	Qualitative Exploratory	To investigate the role played by multiple identity groups in the recovery process. Specifically, how a sense of shared identity with similar others online enhances wellbeing and promotes recovery from *diabulimia* and how important identity networks can be incorporated into the recovery process	Themes: Those with whom one shares a recovery identity can provide psychological resources for recovery; members recognised that other important relationships (including family and friends and health professionals) are also key to recovery; these other group memberships (and the associated identities) can be facilitated through the recovery identity group membership	Findings illustrate the importance of ensuring groups that potentially play a key role in recovery (and positive long‐term health outcomes) should be formally incorporated into the treatment process. This requires clinical interventions to address the ‘relational dimension’ of eating disorders rather than solely focusing on eradicating disordered eating symptoms
Powers and Richter^17^	0	Qualitative Exploratory	To fill a problematic gap in the literature by increasing our understanding of the development and maintenance of an eating disorder among those with T1D	Themes: feeling different (100%), difficulty with control and coping (88%), body image (81%), feelings, mood and emotional state overall and about diabetes and T1DE (69%), and quality of life (44%)	Future research could help to differentiate between typical demands of T1D and those that are unique to persons who develop an eating disorder. Future research should consider developing a brief screening tool that would allow clinical teams to assess eating disorder risk among T1D patients, but do so in a manner that would not suggest either the need to lose weight nor unhealthy practices to manage weight such as omitting insulin
Macdonald and Kan^18^	0	Qualitative Exploratory	To explore the experiential perspective of people with T1DE and that of the HCPs treating them, and to understand the experience of both sides to inform future development of healthcare services	Themes: (1) perceptions surrounding service provision; (2) reflections on the recovery process; (3) the experiential perspective of living with type 1 diabetes and an eating disorder; and (4) support mechanisms	Future research should use a model of a multidisciplinary, collaborative clinical approach to be tested in clinical trials, providing an evidence‐based treatment service to people with T1DE
Staite, Zaremba, Macdonald, et al.^36^	0	Qualitative Exploratory	To perform a qualitative review of online blogs authored by people self‐identifying as having Type 1 diabetes and an eating disorder or ‘diabulimia’	Themes: (1) different aspects of bloggers' relationship with insulin, including motives for omitting insulin, secrecy of insulin omission and perception of control; (2) bloggers' experiences of diabetes complications, and diabetes ketoacidosis in particular, as well as their worries about future complications; (3) strategies for recovery and triggers for relapse, which involved diabetes self‐management and setting up a support system	The deeper‐seated motives behind the insulin manipulation that bloggers described, such as the ‘thrill’, ‘addictive’, ‘experimental’ or ‘secretive’ aspects of insulin omission were novel observations Findings may inform the development of complex interventions to improve biomedical and psychological outcomes in an area where there is limited evidence for effective interventions
Zaremba and Harrison^13^	I	Qualitative Treatment	To test the feasibility of the STEADY intervention, a novel, complex intervention for people with T1DE of mild to moderate severity	The co‐designed protocol, using themes from interviews and focus groups gave feedback for intervention feasibility	The STEADY toolkit will be tested in a 12‐week RCT with an intervention arm or treatment as usual control arm. The trial will include a qualitative process evaluation sub‐study to explore the feasibility, appropriateness and acceptability of STEADY, and will provide an opportunity for refining the STEADY toolkit for future use
Stice and Wisting^11^	II	Pilot RCT Treatment	To evaluate whether the *Body Project* prevention program adapted for young women with type 1 diabetes (*Diabetes Body Project*) reduces eating disorder risk factors and symptoms	*Diabetes Body Project* vs. control showed significantly greater reductions in thin‐ideal internalisation, body dissatisfaction, diabetes distress, diabetes eating pathology, and ED symptoms by post‐test, and greater reductions in diabetes eating pathology and ED symptoms, and greater improvements in QOL by 3‐month follow‐up, which were medium to large effects (*d*'s ranged from −0.43 to −0.90)	A well‐powered RCT is warranted to evaluate this intervention over longer follow‐up
Hennekes and Haugvik^12^	III	RCT; international multicentre trial Prevention	To evaluate a novel ED prevention program in a multinational RCT (*The Body Project*)	Compared with educational controls, participants in the *Diabetes Body Project* showed significant improvements (all *p* < 0.05), with small Cohen's d effect sizes for ED symptoms (*d* = −0.30, 95% CI −0.06, −0.69) (primary outcome), diabetes distress (*d* = −0.42), quality of life (*d* = 0.39) and dietary restraint (*d* = −0.31), and medium effect sizes for diabetes‐specific disordered eating behaviours (*d* = −0.70), body dissatisfaction (*d* = −0.59), and pursuit of thin appearance ideal (*d* = −0.56)	The current article reports only on the post‐intervention effects of the *Diabetes Body Project*. Future research will evaluate whether these effects persist long term and whether the *Diabetes Body Project* significantly reduces future onset of ED, as observed in the standard *Body Project*

**TABLE 2:** Mixed Methods Appraisal Tool (MMAT) quality assessment.

References	Type of study	MMAT questions	Yes	No	Can't tell
Coleman and Caswell^15^	Qualitative	1.1 Is the qualitative approach appropriate to answer the research question?	X		
Notes: 1.2 Open‐ended survey questions were used; therefore, no follow‐up questions could be asked. 1.5 Unable to evaluate due to lack of codebook and full description of analysis					
		1.2 Are the qualitative data collection methods adequate to address the research question?		X	
		1.3 Are the findings adequately derived from the data?	X		
		1.4 Is the interpretation of results sufficiently substantiated by data?	X		
		1.5 Is there coherence between qualitative data sources, collection, analysis and interpretation?			X
Hastings and McNamara^16^	Qualitative	1.1 Is the qualitative approach appropriate to answer the research question?	X		
		1.2 Are the qualitative data collection methods adequate to address the research question?	X		
		1.3 Are the findings adequately derived from the data?	X		
		1.4 Is the interpretation of results sufficiently substantiated by data?	X		
		1.5 Is there coherence between qualitative data sources, collection, analysis and interpretation?	X		
Hennekes and Haugvik^12^	RCT	2.1 Is randomisation properly performed?			X
Notes: 2.1 Randomisation process not described, just ‘293 were randomized’.		2.2 Are the groups compared at baseline?	X		
		2.3 Are there complete outcome data?	X		
		2.4 Are outcome assessors blinded to the intervention provided?	X		
		2.5 Did the participants adhere to the assigned intervention?	X		
Macdonald and Kan^18^	Qualitative	1.1 Is the qualitative approach appropriate to answer the research question?	X		
		1.2 Are the qualitative data collection methods adequate to address the research question?	X		
		1.3 Are the findings adequately derived from the data?	X		
		1.4 Is the interpretation of results sufficiently substantiated by data?	X		
		1.5 Is there coherence between qualitative data sources, collection, analysis and interpretation?	X		
Powers and Richter^17^	Qualitative	1.1 Is the qualitative approach appropriate to answer the research question?	X		
Notes: 1.2 Focus group interviews were conducted; but given the sensitivity of the matter, this may not have been appropriate		1.2 Are the qualitative data collection methods adequate to address the research question?		X	
		1.3 Are the findings adequately derived from the data?	X		
		1.4 Is the interpretation of results sufficiently substantiated by data?	X		
		1.5 Is there coherence between qualitative data sources, collection, analysis and interpretation?	X		
Staite, Zaremba, Macdonald^36^	Qualitative	1.1 Is the qualitative approach appropriate to answer the research question?	X		
		1.2 Are the qualitative data collection methods adequate to address the research question?	X		
		1.3 Are the findings adequately derived from the data?	X		
		1.4 Is the interpretation of results sufficiently substantiated by data?	X		
		1.5 Is there coherence between qualitative data sources, collection, analysis and interpretation?	X		
Stice and Wisting^11^	RCT (pilot)	2.1 Is randomization properly performed?	X		
		2.2 Are the groups compared at baseline?	X		
		2.3 Are there complete outcome data?	X		
		2.4 Are outcome assessors blinded to the intervention provided?	X		
		2.5 Did the participants adhere to the assigned intervention?	X		
Verbist and Condon^14^	Mixed Methods	5.1 Is there an adequate rationale for using a mixed methods design to address the research question?	X		
		5.2 Are the different components of the study effectively integrated to answer the research question?	X		
		5.3 Are the outputs of the integration of qualitative and quantitative components adequately interpreted?	X		
		5.4 Are divergences and inconsistencies between quantitative and qualitative results adequately addressed?	X		
		5.5 Do the different components of the study adhere to the quality criteria of each tradition of the methods involved?	X		
Zaremba and Harrison^13^	Qualitative	1.1 Is the qualitative approach appropriate to answer the research question?	X		
		1.2 Are the qualitative data collection methods adequate to address the research question?	X		
		1.3 Are the findings adequately derived from the data?	X		
		1.4 Is the interpretation of results sufficiently substantiated by data?	X		
		1.5 Is there coherence between qualitative data sources, collection, analysis and interpretation?	X		

### 3.1 Stage 0

Five qualitative studies in Stage 0 held a different aim and purpose: (1) to help providers understand ‘diabulimia’ to make way for future interventions^14^; (2) to incorporate relationships established in peer groups into the treatment process for IRDEB/eating disorders^33^; (3) to emphasise the importance of early identification of those at risk and to develop interventions to mitigate development of an eating disorder^34^; (4) to inform strategies for multidisciplinary care approaches to IRDEB/eating disorders,^33^ involving both people with IRDEB/eating disorders and healthcare providers,^35^ and (5) to analyse experiences of distress by blog authors with type 1 diabetes and an eating disorder or ‘diabulimia’.^36^ While T1D participants reflected on their recovery process, the experiential perspective of living with IRDEB/eating disorders, and their lack of support mechanisms, providers gave personal insight and reflection of their professional role as well as the challenges of working with these dual diagnoses.

In the Stage 0 mixed methods study, the purpose was twofold; first, to examine the predictors of eating disordered behaviours and body image dissatisfaction (*n* = 121); and second, in the same survey, to explore the relationship between type 1 diabetes management and body image through two open‐ended questions (*n* = 98).^32^ Those on the younger end of the mean age (36 ± 6.5) and negative body image explained nearly 60% of disordered eating variance. Satisfaction with body weight and social networking negatively contributed to poor body image (30%). The two qualitative questions were: ‘*Are your body image concerns preoccupying you? If yes, to what extent this affects your everyday life?*’ and ‘*To what extent do you feel that your diabetes treatment affects the way you perceive your body image? Please describe*’. Participants felt they had limited control over their body weight because of insulin requirements (*n* = 48%) and they did not want visible medical equipment (*n* = 7%). Because of its focus on body image and negative impacts of media, the recommended future direction is to develop prevention programs that target increases in body image satisfaction in type 1 diabetes.

### 3.2 Stage I

Uniquely, a qualitative study in Stage I used a participatory action design in ‘Safe management of people with type 1 diabetes and eating disorders study’ (STEADY).^31^ The process of developing workshops was co‐designed through interviews and focus groups with health care professionals (*n* = 22) and people who had (or have had) a history of IRDEB/eating disorders (*n* = 14). The purpose of the workshops was to co‐develop content to be included in a toolkit of cognitive behavioural therapy (CBT) techniques to help treat IRDEB/eating disorders. In workshop one, adults with IRDEB/eating disorders discussed how they believed ‘insulin equals fat’, and that ‘insulin omission is my magic weight loss tool’, with a recommendation to focus on insulin titration and the need for taking small steps. In workshop two, the fear of hypoglycaemia and ‘hypos force me to eat’, were addressed. It became clear from the first two workshops that individualised and stepwise care plans were essential for recovery, and a manual would not be sufficient; thus, the third workshop was devoted to tailoring the specific concerns and requirements of the individual so that presentations of IRDEB/eating disorders could be chosen by the therapist for each patient's needs. This qualitative work will be tested for feasibility in an RCT into the STEADY intervention group or treatment as usual control (*n* = 40).^37^

### 3.3 Stage II

The *Diabetes Body Project*, a 6‐week, 1‐h, general eating disorder virtual programme for those with type 1 diabetes, was first developed in a pilot RCT with women ages 15–30 (mean = age 22.06 [4.66] in control, mean = age 23.50 [3.83] in intervention at baseline).^29^ Eligible participants completed the baseline assessment (diagnostic interview, surveys and 2‐week wear of a continuous glucose monitor). After the baseline assessment, participants were randomly assigned to virtual *Diabetes Body Project* groups co‐led by a clinician and a peer educator with type 1 diabetes (*n* = 30) or to a control condition in which they watched educational videos about eating disorders and type 1 diabetes (*n* = 25). The *Diabetes Body Project* participants showed significantly greater improvements in eating disorder symptoms and behaviours, pursuit of the thin appearance, body dissatisfaction and dietary restraint as well as diabetes distress and diabetes‐related quality of life than control participants. Recommendations for future research suggest utilising the qualitative feedback from this trial to refine the *Diabetes Body Project* and conduct a fully powered trial that evaluates the effects on these continuous outcomes and tests whether this intervention significantly reduces future onset of IRDEB/eating disorders over long‐term follow‐up, which is described in Stage III.

### 3.4 Stage III

Although studies of T1DE have risen in publication, the *Diabetes Body Project* was the only completed ‘real‐world’ efficacy‐testing RCT since 2014 focused on young women with type 1 diabetes and eating disorders/disordered eating.^30^ In this study, researchers tested a fully powered, multi‐site RCT that included adults up to age 35 (mean = age 25.2 [5.43] in control, mean = 24.8 [5.58] in intervention at baseline). Similar to the pilot RCT, significant improvements were demonstrated for eating disorder symptoms (primary outcome), diabetes distress, quality of life and dietary restraint with medium effect sizes for diabetes‐specific disordered eating behaviours, body dissatisfaction and pursuit of thin appearance. This study is being tested longitudinally to follow‐up with participants over a 2‐year period to determine whether improvements persist. Implications for future research recommend testing on a broad scale.

## 4 DISCUSSION

The studies in this systematic review explicitly stated that the next step in development of the research is continuing to the next stage, even though they did not refer to the NIH Stage Model directly.^29^, ^30^, ^31^, ^32^, ^35^, ^37^ By discussing the need for further research in their articles, authors made a specific recommendation for more studies to advance through testing in clinical trials,^32^, ^35^ in a feasibility RCT,^31^ in a well‐powered RCT,^29^ and in longitudinal studies^30^ Emphasis on scalability are significantly influencing intervention development in aging,^28^ with a goal of increasing the use of the NIH Stage Model in all areas of basic and qualitative research, efficacy, effectiveness and implementation trials.

With the limited research that is available, and only one completed RCT, there are multiple opportunities for continued research in IRDEB/eating disorders. Only one project from this review demonstrated how research can advance from an adaptation of an intervention^29^ to an RCT^30^ (*The Diabetes Body Project*), and one conducted a pilot study RCT for feasibility^31^ (STEADY). There is a need to investigate the effectiveness of interventions with Stage II and Stage III trials, keeping in mind the unique needs and conditions under which IRDEB/eating disorder symptoms emerge.

In Stage II, specifically, researchers can focus on what works in the intervention to accentuate the mechanisms that cause meaningful changes (and eliminate those that don't make a difference) for replication. Tackling implementation‐related issues, such as scalability, usability, ease of delivery and training of real‐world providers to deliver the intervention, must be considered in these early stages of the behavioural intervention to move research from efficacy (Stage III) to effectiveness (Stage IV). Researchers who have conducted work in earlier stages, such as in qualitative work (Stage 0) or in pilot studies (Stage I), would benefit from creating a long‐term plan early in the process to incorporate future studies at higher stages in the NIH Model.

We also reviewed articles that did not have control/comparison groups and were not included in the review to determine whether there were published protocol or evidence that work was continuing. In cases where it was unclear as to the status of research evidence, authors of the manuscripts were contacted for clarification. One single arm pilot study (Merwin et al.) was identified (from *k* = 72) as having an RCT underway in a registered protocol (ClinicalTrials.gov identifier ID NCT0554070).^38^ This trial compared a tailored Acceptance and Commitment Therapy (ACT) intervention for type 1 eating disorders (iACT) to Supportive Diabetes Counseling. iACT is based on the premise that ED symptoms emerge as individuals attempt to cope with emotional distress related to T1D, eating and weight. In the original pilot trial consisting of a sample of mostly white women with bulimia nervosa and binge eating disorders, participants (*n* = 23) reported increased psychological flexibility with diabetes‐related thoughts/feelings, and less obstruction and greater progress in pursuing personal values. There were large effects for change in ED symptoms, diabetes self management and diabetes distress from baseline to end‐of‐treatment (Cohen's *d* = 0.90–1.79). HbA1c also improved, but the *p*‐value did not reach statistical significance, *p* = 0.08. The findings provided preliminary support for tailored ACT to improve outcomes for T1DE leading to the current Stage II randomised controlled trial. This is another example of how researchers can build upon evidence in the NIH Stage Model.

The use of a systematic review methodology is a strength of this research, as well as the use of Covidence software to document decisions made regarding inclusion/exclusion criteria. Methods of searching, inclusion of sources and data charting were systematic, rigorous and pre‐registered, thus minimising potential bias in the gathering and analysis of evidence relevant to the review of IRDEB/eating disorders in type 1 diabetes. A strength was using the NIH Model as a synthesising and categorising framework in discussing future research and recommendations for the area of research. Limitations of this study included: (1) We did not report experimental research that did not have a control/comparison group, potentially limiting our use of the NIH Stage Model. (2) We reviewed articles in the last 10 years of systematic reviews that fell within the timeframe (e.g., Clery et al.),^20^ which referenced articles prior to 2014 that may contribute to the literature, such as the first study conducted in this area.^39^ In particular, earlier articles on psychoeducation techniques and eating disorders,^40^, ^41^ CBT therapies for binge‐eating,^42^ mental health,^43^ or several articles by Goebel‐Fabbri^44^, ^45^, ^46^, ^47^, ^48^, ^49^ may have been useful for this discussion; however, the time‐frame (2014–2024) was chosen to collect the most up‐to‐date research in this area. (3) It was beyond the scope of this study to include a primary biomedical outcome; rather, we included studies with only a primary outcome of psychological factors. Additional research could investigate the impact of psychological interventions of type 1 diabetes or qualitative research with HbA1c, time in range, or weight changes as primary outcomes.

Based on the literature and authors' recommendations for future research, these are priorities for research in the field of IRDEB/eating disorders.

1. Continue with the research that suggests an advancement in the NIH Stage Model.
2. None of the studies in the review included interventions with the healthcare professional; all studies were directed towards patient activation for improvements. (One qualitative study did include both IRDEB/eating disorder adults and providers to develop an intervention.)^31^ Helping HCPs recognise IRDEB/eating disorders early is key to prevention of escalating symptoms. Interventions for HCPs also could include experiential modelling with standardised patients to practice real‐time communication with patients when IRDEB/eating disorders is recognised.
3. As recognised,^30^ conduct longitudinal studies (long‐term cohort) of prevention, early at‐risk detection and early intervention to shift the trajectory of IRDEB/eating disorders. At least partial focus of these interventions is educating type 1 diabetes patients that gaining weight due to insulin after diagnosis is normal, and that gaining weight is a sign that the body is recovering from being malnourished.

In conclusion, there is much work to be done, both qualitatively and quantitatively at all stages of behavioural intervention. From this review, it is clear that the research trajectory needs to be established for behavioural interventions to plan for all stages of the NIH Stage Model. More RCTs with qualitative data at Stage 0/1 and longitudinal trials of IRDEB/eating disorders at stages II and beyond need to be undertaken, supported by funding mechanisms for researchers to continue to advance their research forward in the NIH Stage Model. As behavioural and social science researchers, we need to address the urgent need for evidence‐based, implementable interventions to help the growing number people of type 1 diabetes and negative psychological outcomes, such as IRDEB and eating disorders.

## FUNDING INFORMATION

1‐SRA‐2025‐1613‐S‐B/Breakthrough T1D/United States.

## CONFLICT OF INTEREST STATEMENT

The authors report no conflicts of interest related to this project.

## Supporting information


**Table S1.** Search terms for systematic review.

**Table S2.** Qualitative extractions.

**Table S3.** Quantitative extractions.

**Table S4.** Mixed methods extractions.
