# Supplementary material for: Psychological research in eating disorders/disordered eating in type 1 diabetes: A systematic review using the NIH stage model for behavioural intervention development
Source: Diabet Med. 2026 May 21;43(9):e70369. doi: 10.1111/dme.70369 (PMC13613988; doi:10.1111/dme.70369)
Supplement: Supplementary file 1 [file DME-43-e70369-s001.docx]

*Supplemental Table S1 Search Terms*

**COCHRANE**
Diabetes AND Distress, “disordered eating”

**CINAHL**
MH "Diabetes Mellitus, Type 1+" AND (MH "Disordered Eating Behavior" OR MH "Eating Disorders+" OR MH "Binge Eating Disorder" OR MH "Eating Disorders Management (Iowa NIC)" OR MH "Eating Behavior+" OR MH "Eating") AND MH "Psychological Distress" AND “Intervention" OR MH "Psychosocial Intervention"

**PSYCINFO**
diabetes distress T/A, “diabetes type 1” AND intervention Limit journals, ten years, peer reviewed adults

**SCOPUS**
diabetes and distress NOT caregiver NOT parent NOT type 2 limit 10 years, adult, English

**WEB OF SCIENCE**
diabetes distress (Topic) and Type 1 Diabetes (Title) and intervention (All Fields) and Review Article or Article (Document Types) and English (Languages) and Article or Review Article (Document Types) and Health Care Sciences Services or Psychology Developmental or Psychiatry or Nursing or Medicine General Internal or Psychology Clinical or Medicine Research Experimental or Public Environmental Occupational Health or Endocrinology Metabolism or Nutrition Dietetics or Multidisciplinary Sciences or Psychology or Psychology Multidisciplinary or Social Sciences Interdisciplinary or Family Studies or Health Policy Services or Social Sciences Biomedical or Behavioral Sciences or Neurosciences or Sociology or Primary Health Care or Physiology (Web of Science Categories)

**MEDLINE (PUBMED)**
((diabetes distress[Title/Abstract]) AND (("diabetes type 1"[Title/Abstract]) OR ("Diabetes Mellitus, Type 1"[Mesh]))) AND ((intervention[Title/Abstract]) OR ("Psychosocial Intervention"[Mesh])) 71 results

("diabetes distress"[Title/Abstract] AND "type 1"[Title/Abstract] AND "intervention"[Title/Abstract]) AND ((y_10[Filter]) AND (english[Filter]) AND (alladult[Filter]))

(((intervention[Title/Abstract]) OR ("Psychosocial Intervention"[Mesh])) AND ((anxiety[Title/Abstract]) OR ("Anxiety"[Mesh]))) AND (("diabetes type 1"[Title/Abstract]) OR ("Diabetes Mellitus, Type 1"[Mesh])) 27 results

((("diabetes type 1"[Title/Abstract]) OR ("Diabetes Mellitus, Type 1"[Mesh])) AND ("Depression"[Mesh] OR "Depressive Disorder"[Mesh])) AND ((intervention[Title/Abstract]) OR ("Psychosocial Intervention"[Mesh])) 28 results

((("diabetes type 1"[Title/Abstract]) OR ("Diabetes Mellitus, Type 1"[Mesh])) AND ((intervention[Title/Abstract]) OR ("Psychosocial Intervention"[Mesh]))) AND ((diabulimia[Title/Abstract]) OR ("Diabulimia"[Mesh])) 3 results

((anorexia[Title/Abstract] OR Bulimia[Title/Abstract] OR "eating disorder"[Title/Abstract]) AND (("diabetes type 1"[Title/Abstract]) OR ("Diabetes Mellitus, Type 1"[Mesh]))) AND ((intervention[Title/Abstract]) OR ("Psychosocial Intervention"[Mesh])) 11 results

**EMBASE**
#11. 'type 1 diabetes' AND 'intervention study'/exp

#10. ((('type 1 diabetes':ab,ti AND intervention:ab,ti AND (anxiety:ab,ti OR depression:ab,ti OR disorder:ab,ti)) AND ([adult]/lim OR [aged]/lim OR [middle aged]/lim OR [young adult]/lim)) AND (2014:py OR 2015:py OR 2016:py OR 2017:py OR 2018:py OR 2019:py OR 2020:py OR 2021:py OR 2022:py OR 2023:py OR 2024:py)) AND ([adult]/lim OR [aged]/lim OR [middle aged]/lim OR [young adult]/lim)

#9. (('type 1 diabetes':ab,ti AND intervention:ab,ti AND (anxiety:ab,ti OR depression:ab,ti OR disorder:ab,ti)) AND ([adult]/lim OR [aged]/lim OR [middle aged]/lim OR [young adult]/lim)) AND (2014:py OR 2015:py OR 2016:py OR 2017:py OR 2018:py OR 2019:py OR 2020:py OR 2021:py OR 2022:py OR 2023:py OR 2024:py)

#8. ('type 1 diabetes':ab,ti AND intervention:ab,ti AND (anxiety:ab,ti OR depression:ab,ti OR disorder:ab,ti)) AND ([adult]/lim OR [aged]/lim OR [middle aged]/lim OR [young adult]/lim)

#7. 'type 1 diabetes':ab,ti AND intervention:ab,ti AND (anxiety:ab,ti OR depression:ab,ti OR disorder:ab,ti)

#6. ((('insulin dependent diabetes mellitus'/exp OR 'insulin dependent diabetes mellitus') AND ('intervention study'/exp OR 'intervention study') AND distress:ab,ti) AND ([adult]/lim OR [aged]/lim OR [middle aged]/lim)) AND (2014:py OR 2015:py OR 2017:py OR 2018:py OR 2019:py OR 2020:py OR 2021:py OR 2022:py OR 2023:py OR 2024:py)

#5. (('insulin dependent diabetes mellitus'/exp OR 'insulin dependent diabetes mellitus') AND ('intervention study'/exp OR 'intervention study') AND distress:ab,ti) AND ([adult]/lim OR [aged]/lim OR [middle aged]/lim)

#4. ('insulin dependent diabetes mellitus'/exp OR 'insulin dependent diabetes mellitus') AND ('intervention study'/exp OR 'intervention study') AND distress:ab,ti

#3. distress:ab,ti 230,953 10

#2. 'intervention study'/exp OR 'intervention study'

#1. 'insulin dependent diabetes mellitus'/exp OR 'insulin dependent diabetes mellitus'

#5. diabulimia:ab,ti AND intervention:ab,ti

#4. (('diabetes type 1':ab,ti AND (anxiety:ab,ti OR depression:ab,ti OR disorder:ab,ti)) AND ([adult]/lim OR [aged]/lim)) AND (2014:py OR 2015:py OR 2016:py OR 2017:py OR 2018:py OR 2019:py OR 2020:py OR 2021:py OR 2022:py OR 2023:py OR 2024:py)

#3. ('diabetes type 1':ab,ti AND (anxiety:ab,ti OR depression:ab,ti OR disorder:ab,ti)) AND ([adult]/lim OR [aged]/lim)

#2. 'diabetes type 1':ab,ti AND (anxiety:ab,ti OR depression:ab,ti OR disorder:ab,ti)

#1. 'diabetes type 1':ab,ti AND 'diabetes 7 distress':ab,

#5 diabulimia:ab,ti AND intervention:ab,ti
#4 #3 AND (2014:py OR 2015:py OR 2016:py OR 2017:py OR 2018:py OR 2019:py OR 2020:py OR 2021:py OR 2022:py OR 2023:py OR 2024:py)
#3 #2 AND ([adult]/lim OR [aged]/lim)
#2 'diabetes type 1':ab,ti AND (anxiety:ab,ti OR depression:ab,ti OR disorder:ab,ti)
#1 'diabetes type 1':ab,ti AND 'diabetes distress':ab,ti
